# The relationship between COVID-19 and opioid-related emergency department visits in Alberta, Canada:an interrupted time series analysis

**DOI:** 10.24095/hpcdp.45.9.01

**Published:** 2025-09

**Authors:** Kelsey A. Speed, Hauwa Bwala, Nicole D. Gehring, Marawan Ahmed, Kathryn Dong, Parabhdeep Lail, Shanell Twan, Gillian Harvey, Patrick McLane, Ginetta Salvalaggio, T. Cameron Wild, Klaudia Dmitrienko, Joshua Hathaway, Elaine Hyshka

**Affiliations:** 1 School of Public Health, University of Alberta, Edmonton, Alberta, Canada; 2 Quantitative Solutions, Applied Pharmaceutical Innovation, Edmonton, Alberta, Canada; 3 Department of Emergency Medicine, University of Alberta, Edmonton, Alberta, Canada; 4 Department of Medicine, University of Calgary, Calgary, Alberta, Canada; 5 The Alberta Alliance Who Educate and Advocate Responsibly (AAWEAR), Edmonton, Alberta, Canada; 6 Department of Art and Design, University of Alberta, Edmonton, Alberta, Canada; 7 Emergency Strategic Clinical Network, Alberta Health Services, Alberta, Canada; 8 Department of Family Medicine, University of Alberta, Edmonton, Alberta, Canada; 9 Alberta Health Services, Edmonton, Alberta, Canada

**Keywords:** COVID-19, opioid use, emergency medicine, public health, people who use drugs, substance use, drug overdose

## Abstract

**Introduction:**

Emergency departments (EDs) are important health care access points for people who use drugs (PWUD), but little is known about whether the onset of the COVID-19 pandemic was associated with changes in opioid-related emergency presentations. We investigated whether (1) the onset of the COVID-19 pandemic was associated with any change in average rates of opioid-related ED visits in Alberta; and (2) this varied across regions with different COVID-19 case rates.

**Methods:**

We conducted maximum-likelihood interrupted time series analyses to compare opioid-related ED visits during the “prepandemic period” (3 March 2019–1 March 2020) and the “pandemic period” (2 March 2020–14 March 2021).

**Results:**

There were 8883 and 11 657 opioid-related ED visits during the prepandemic and pandemic periods, respectively. The onset of the COVID-19 pandemic was associated with an increase in opioid-related ED visits (Edmonton: IRR = 1.37, 95% CI: 1.30–1.44, *p* < 0.05; Calgary: IRR = 1.14, 95% CI: 1.07–1.20, *p* < 0.05; Other health zones: IRR = 1.14, 95% CI: 1.07–1.21, *p* < 0.05). Changing COVID-19 case counts did not correspond with changing rates of opioid-related ED visits across regions.

**Conclusion:**

The increase in opioid-related ED visits associated with the onset of the COVID-19 pandemic was unrelated to COVID-19 case prevalence in Alberta.

**Highlights**

- The COVID-19 pandemic exacerbated harms among people who use drugs, including through capacity reductions in health and social services.
- We compared opioid-related emergency department visits in the year before the pandemic (3 March 2019 to 1 March 2020) with those in the first year of the pandemic (2 March 2020 to 14 March 2021).
- The onset of the COVID-19 pandemic was associated with an increase in opioid-related emergency department visits, which was unrelated to the prevalence of COVID- 19 cases.
- Research is needed to determine how to best support people who use drugs during pandemics and other emergencies in the future.

## Introduction

The COVID-19 pandemic coincided with Canada’s ongoing drug poisoning crisis to exacerbate risks for people who use drugs (PWUD).^1^,^2^ This was particularly apparent in Alberta, where these dual public health crises resulted in significant mortality.^3^-^5^ Border closures and disrupted supply chains led to increased contamination, toxicity and costs of illegal drugs.^6^-^9^ Social isolation and financial and psychological strain may have resulted in people using substances as a coping mechanism.^6^,^8^-^10^ Non-urgent health and social services for PWUD reduced their capacity to accommodate enhanced infection prevention and control measures,^11^ which limited access to supervised consumption services,^3^ primary care,^12^ withdrawal management and treatment facilities,^13^ sterile drug supplies, naloxone kits and drug-checking services.^14^,^15^ As a result, more people may have been using drugs alone or in other unsafe conditions for longer periods of time, increasing their risk of drug poisoning and infections^16^ and their likelihood of requiring emergency care.^17^

The COVID-19 pandemic and the associated infection prevention and control measures resulted in changes in the numbers of people seeking emergency department (ED) care and in how ED care was delivered. The overall number of people seeking ED care decreased soon after the onset of the pandemic,^18^ likely because of concerns about in-hospital transmission of COVID-19, fewer injuries as a result of stay-at-home orders and reductions in non-COVID-19–related in-hospital procedures.^19^ ED personnel also altered how they triaged and engaged with PWUD and decreased prescribing of opioid agonist treatment (OAT),^20^ shifted appointments with ancillary support staff (e.g. counsellors) from in-person to remote, which hindered rapport-building, and reduced referrals to community-based services following discharge.^14^ This is particularly salient as EDs are key health care access points for PWUD, and health care providers in the ED can mitigate future opioid-related harm by initiating OAT,^21^-^23^ distributing naloxone and providing referrals to community-based services.^24^

It is unclear how the COVID-19 pandemic and its associated impacts on EDs affected opioid-related emergency presentations. We conducted this study to determine (1)whether the onset of the COVID-19 pandemic was associated with a change in the average rates of opioid-related ED visits in Alberta; and (2) whether changes in average rates of opioid-related ED visits varied across regions with different COVID-19 case rates. We hypothesized (1) that the onset of the COVID-19 pandemic led to an increased incidence rate of opioid-related ED visits; and (2) that there is an association between the trends in COVID-19 case counts and the rate of opioid-related ED visits across regions.

## Methods

***Ethics approval***

The University of Alberta Research Ethics Board 3: Health Research Ethics Board – Health Panel provided ethics approval (Pro00103203) and a waiver of consent; informed consent from participants was not required as we extracted anonymized administrative health data.

***Study design***

We conducted an interrupted time series analysis to test whether the incidence of opioid-related ED visits in Alberta changed during the first year of the COVID-19 pandemic (referred to as the “pandemic period,” 2 March 2020 to 14 March 2021) compared to before the pandemic (referred to as the “prepandemic period,” 3 March 2019 to 1March 2020) using counts of opioid-related ED visits over 2-week periods. Interrupted time series analyses are commonly used in observational public health studies to make pre–post comparisons by adjusting for pre-existing time trends, seasonality and other time-varying confounders.^25^ The pandemic period was designated according to the implementation of Alberta’s acute care COVID-19 response protocols (at the beginning of March 2020), and we created equal-length prepandemic and pandemic periods (27data points of 2-week periods each) for pre–post analysis.

Alberta is covered by a single provincial health authority (Alberta Health Services)^26^ that is made up of five defined health zones: North, Edmonton, Central, Calgary and South.^27^ Each health zone varies in population and geographic size; the Edmonton and Calgary health zones, which cover metropolitan areas, have higher populations and smaller geographic areas than the North, Central and South health zones, which are more rural. There were 105 EDs (including community ambulatory sites) across the province with data available for the study period (34 in North health zone, 13 in Edmonton health zone, 29 in Central health zone, 18 in Calgary health zone and 11 in South health zone). While there are other EDs in the province, not all had data available for the study period. We merged the North, South and Central health zones to create the “Other health zones” variable because of the limited number of data points available in each of these less-populated health zones.

***Data source***

We extracted opioid-related ED data (i.e. raw counts of patients) for the period 3March 2019 to 14 March 2021 from the National Ambulatory Care Reporting System (NACRS). The NACRS collects health records on all ED visits in Alberta.^28^ We included all ICD-10–coded diagnoses of poisoning related to opium (T40.0), other opioids (T40.2) and other/unspecified narcotics (T40.6) and of mental and behavioural disorders due to the use of opioids (F11.0–F11.9). We retrieved demographic data (sex, health zone) for all ED visits with these diagnoses.

***Data analysis***

We evaluated trends in ED visits for opioid-related illnesses using 2-week time periods over 2 years using maximum-likelihood interrupted time series analysis for counts. We tested for correlations between variables before analysis. Negative binomial regression models (as a result of the non-normal distribution of the data and overdispersion) were then fitted to test the two study hypotheses. We modelled rates directly with a log-linear statistical model by including counts as the dependent variable and controlled for underlying trends, such as seasonality, by including 2-week time periods in the model as a dummy variable. All hypothesis tests used a significance level (alpha) of 0.05. We performed regression diagnostics postestimation, including quasi-likelihood information criteria to assess goodness of fit. Statistical analyses were performed using Stata version 17 (StataCorp LLC, College Station, TX, US) using the “nbreg” and “margins” commands.

## Results

There were 20 540 opioid-related ED visits across Alberta during the study period: 8883 (43.2%) visits during the prepandemic period and 11 657 (56.9%) visits during the pandemic period. During the entire study period, 7934 (38.6%) of the opioid-related ED visits occurred in the Edmonton health zone, 7120 (34.7%) in the Calgary health zone and 5486 (26.7%) in the Other health zones. Based on Alberta Health Services’ definition of sex (the sex documented on the government-issued identification presented at the time of registration), 12 338 (60.1%) of the patients were male and 8202 (39.9%) were female.

**Figure 1:**
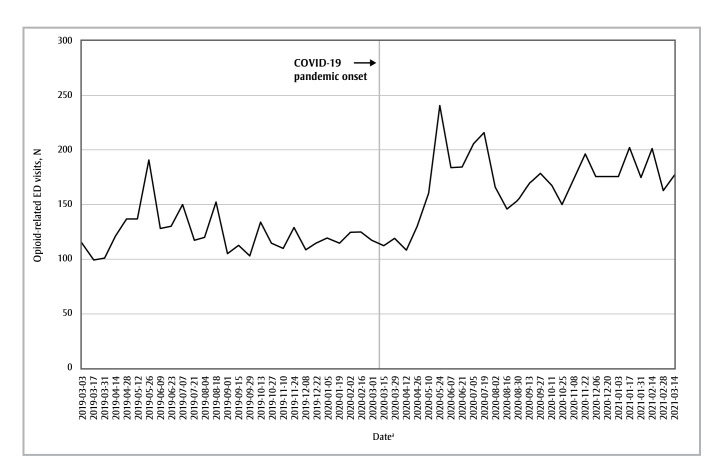
Number of opioid-related ED visits in each 2-week period from 3 March 2019 to 1 March 2020 (the “prepandemic period”) and from 2 March 2020 to 14 March 2021 (the “pandemic period”), Edmonton health zone, Alberta, Canada

**Abbreviation: **ED, emergency department. 

^a^ Start date of each 2-week data-gathering period.

In the Edmonton health zone, the pandemic period was associated with an increased incidence rate of opioid-related ED visits compared to the prepandemic period (incidence rate ratio [IRR] = 1.37; 95% confidence interval [CI]: 1.30–1.44; *p*< 0.05) (Figure 1).

**Figure 2:**
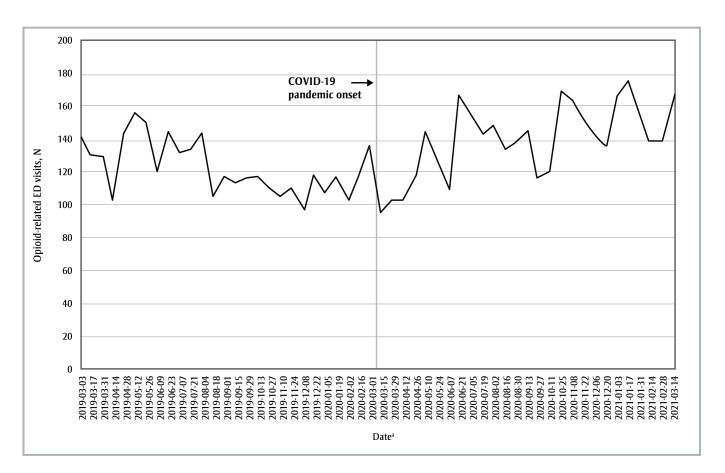
Number of opioid-related ED visits in each 2-week period from 3 March 2019 to 1 March 2020 (the “prepandemic period”) and from 2 March 2020 to 14 March 2021 (the “pandemic period”), Calgary health zone, Alberta, Canada

**Abbreviation: **ED, emergency department. 

^a^ Start date of each 2-week data-gathering period.

**Figure 3:**
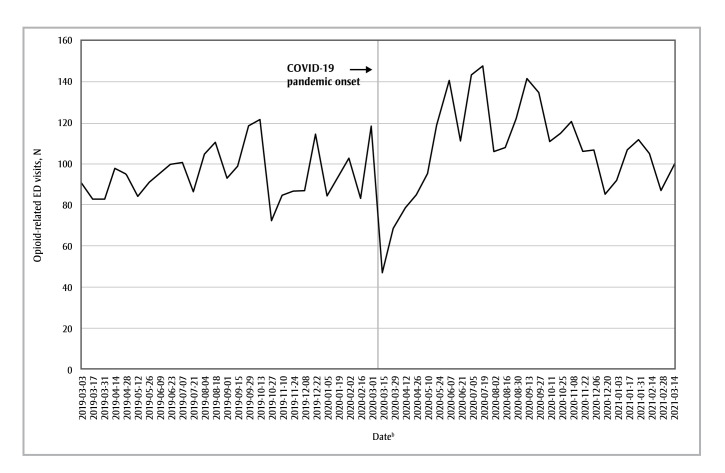
Number of opioid-related ED visits in each 2-week period from 3 March 2019 to 1 March 2020 (the “prepandemic period”) and from 2 March 2020 to 14 March 2021 (the “pandemic period”), Other health zones, Alberta, Canada

**Abbreviation:** ED, emergency department. 

^a^ Alberta Health Services North, South and Central health zones were combined into the “Other health zones” variable because of the limited number of data points available in each of these less-populated health zones. 

^b^ Start date of each 2-week data-gathering period. 

The pandemic period was also associated with increased incidence rates of opioid-related visits in the Calgary health zone (IRR = 1.14; 95% CI: 1.07–1.20; *p* < 0.05) and the Other health zones (IRR = 1.14; 95% CI: 1.07–1.21; *p* < 0.05), albeit to lesser extents than in the Edmonton health zone (Figures 2 and 3).

**Figure 4:**
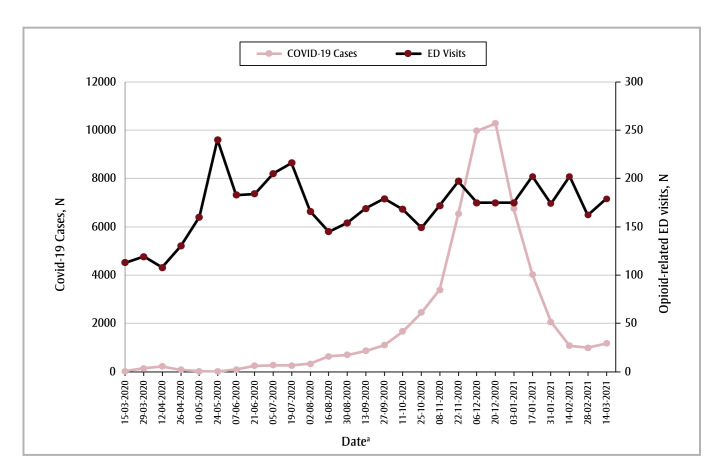
Number of COVID-19 cases and opioid-related ED visits in each 2-week period from 15 March 2020 to 14 March 2021, Edmonton health zone, Alberta, Canada

**Abbreviation:** ED, emergency department. 

^a^ Start date of each 2-week data-gathering period. 

**Figure 5:**
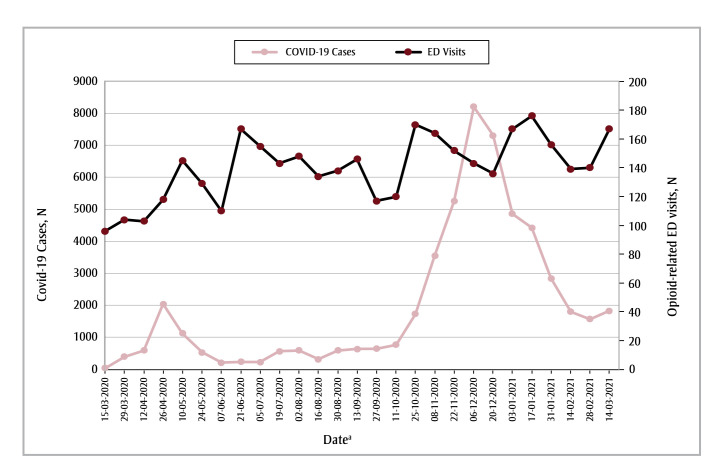
Number of COVID-19 cases and opioid-related ED visits in each 2-week period from 15 March 2020 to 14 March 2021, Calgary health zone, Alberta, Canada

**Abbreviation:** ED, emergency department. 

^a^ Start date of each 2-week data-gathering period.

The trends in COVID-19 case counts between March 2020 and March 2021 were not associated with the rates of opioid-related ED visits in the Edmonton health zone (IRR = 1.00; 95% CI: 0.99–1.00; *p*= 0.23) or the Calgary health zone (IRR= 1.00; 95% CI: 1.00–1.00; *p* < 0.05) (Figures 4 and 5). Because of the variability in the number of COVID-19 cases in the Other health zones, we were unable to model this relationship for these rural zones.

## Discussion

The onset of the COVID-19 pandemic was associated with an increase in the rate of opioid-related ED visits across the Edmonton, Calgary and Other health zones, despite overall reductions in ED visits in Alberta for all conditions from 2019 to 2020.^29^ The Edmonton health zone had the highest incidence rate of opioid-related visits during the COVID-19 pandemic. The regression model also revealed an increase in the mean absolute counts of ED visits for opioid-related concerns during the COVID-19 pandemic period in Alberta. However, COVID-19 case counts were not associated with rates of opioid-related ED visits in either the Edmonton or Calgary health zones.

Our findings align with those from studies conducted in the United States,^30^ and provide much-needed Canadian context into the complex intersection between the COVID-19 pandemic and the drug poisoning crisis. Given the distinct dynamics driving infectious disease transmission and drug poisoning harms, direct comparisons between the public health responses to each crisis are problematic. Nonetheless, governments can mobilize resources and expertise quickly in response to a public health crisis. Efforts to mitigate the impacts of COVID-19—including public health orders (e.g. mask and isolation mandates),^31^ a coordinated vaccination strategy^32^ and the implementation of surge capacity in intensive care units^33^—were instituted early and maintained for 2years. The response to the ongoing drug poisoning crisis has differed in scope and pace, despite the 8282 opioid poisoning deaths that occurred in Alberta between January 2016 and August 2023.^3^ Comparable efforts to mitigate the impacts of the drug poisoning crisis could include governments promoting widespread access to evidence-based services and treatment options (e.g. supervised consumption services, OAT) through pharmacies and temporary or mobile clinics, for example.

In addition, the response to these two intertwined public health emergencies has been largely independent of each other, despite the potential for exacerbated harms for PWUD during emergencies and periods of substantial social change such as the COVID-19 pandemic.^6^ Indeed, the swift and significant increase in opioid-related harms following the onset of the COVID-19 pandemic serves as an important lesson for future public health emergencies, highlighting the importance of paying particular attention to the needs of PWUD when planning for future public health crises in the context of the ongoing drug poisoning crisis. Ensuring the best health outcomes for PWUD requires that governments swiftly implement cohesive evidence-based responses that consider the interdependence of both public health crises.

Several factors may have played a role in the larger increase in opioid-related ED visits in the Edmonton health zone compared to the Calgary and Other health zones; here, we discuss two possible factors. First, geographic differences in the illegal drug supply may have led to different rates of acute opioid toxicity in each of the health zones: provincial toxicology data show the presence of carfentanil in 20% and 54% of acute drug toxicity deaths related to any opioid in Edmonton in 2020 and 2021, respectively, and in only 1% to 5% of these deaths in Calgary during the same periods.^3^ Carfentanil, a synthetic opioid that is particularly potent compared to other opioids (e.g. heroin, fentanyl),^34^ has been associated with increased rates of acute drug toxicity and deaths in the United States.^35^

Second, the number of people experiencing homelessness in Edmonton increased from 1971 in 2018^36^ to more than 3000 in 2022,^37^ while this population remained relatively stable in Calgary (2911 in 2018^38^ to 2782 in 2022^39^). Experiencing homelessness has been associated with increased opioid-related ED visits and hospitalizations before^40^,^41^ and during^42^ the COVID-19 pandemic. Moreover, opioid poisoning deaths in public spaces in Alberta increased during the pandemic,^3^ which may suggest greater risk of severe opioid outcomes requiring immediate ED care among individuals experiencing homelessness. It is possible that the difference in rates of homelessness in Edmonton and Calgary could further contribute to the variance in rates of opioid-related ED visits found in this study.

Future research should explore whether changes in acute care settings (e.g. new personal protective equipment requirements^43^), clinician and health care worker burnout^44^ or other consequences of the COVID-19 pandemic influenced the provision of patient-centred and evidence-based interventions for ED patients with opioid-related concerns.

***Strengths and limitations***

The present findings document an increase in opioid-related ED visits during the pandemic period, but our study has limitations that may affect the interpretation of these findings. First, the limited number of data points in the North, South and Central health zones precluded a more nuanced analysis of the relationship between the COVID-19 pandemic and opioid-related ED visits in geographic areas outside of the two major cities in the province.

Second, this analysis is based on ICD-10 codes as recorded in the administrative data. These codes may be applied inconsistently (e.g. because of misclassification bias, misdiagnosis, unclear or misinterpreted clinical notes, and changes in diagnostic or record-keeping practices within EDs^45^) and may not reflect the true number of ED visits related to opioid use.

Third, we were unable to account for the volatility in the drug supply across the different health zones during our study period due to a dearth of reliable publicly available information on the drug supply.

Finally, Alberta surveillance data from 2016 to 2022 show that emergency medical services responses to opioid-related emergencies were highest among people aged 20 to 39 years, while hospitalizations were highest among people aged 60 years or older (except in 2021, when people aged 30 to 39 years had the highest percentage of hospitalizations).^46^ However, because we could only collect the mean age of all the visitors during each 2-week period, as opposed to the individual age of each visitor, we do not discuss age at the time of ED visits and are unable to confirm whether the data included in our study are consistent with these trends. Future research that collects individual-level age data could show important age-specific trends in opioid-related ED visits and the demographics most affected by opioid-related emergencies.

## Conclusion

Canada has had to contend with two concurrent public health crises since 2020. The COVID-19 pandemic and drug toxicity crisis have strained the health care system and resulted in unprecedented numbers of deaths. During the first year of the COVID-19 pandemic, harms for PWUD were exacerbated and opioid-related ED visits increased across all health zones in Alberta. The rate of opioid-related ED visits was more strongly associated with the onset of the pandemic in the Edmonton health zone than in the Calgary or Other health zones. Further research is needed to determine whether the COVID-19 pandemic influenced the quality of ED care received by care-seeking PWUD and how to best support PWUD receiving the best possible care in EDs during future pandemics and other emergencies.

## Acknowledgements

We gratefully acknowledge the valuable contributions of Lexis R. Galarneau, who helped with manuscript formatting, and Dr. May Mrochuk (knowledge user), Dr. Katherine Rittenbach (co-applicant) and Shelly Vik (collaborator) for their roles in acquiring the Canadian Institutes of Health Research (CIHR) operating grant that funded this study. Administrative Data Analytic Support was provided by the Canadian Research Initiative in Substance Misuse (CRISM) Prairie Node via the CRISM-AHS Advancement in Analytics in Addiction partnership.

## Funding

This project was funded by a CIHR Operating Grant on Developing Innovative Adaptations of Services/Delivery under grant 448953. This research was also undertaken, in part, thanks to funding from the Canada Research Chairs Program via a Tier II Canada Research Chair in Health Systems Innovation to Elaine Hyshka.

## Conflicts of interest

EH and GS have received grant funding from the Royal Alexandra Hospital Foundation and the CIHR. EH has also received grant funding from the Canadian Public Health Association, Health Canada and Alberta Health Services. She has also received a contributor fee from Alberta Views Magazine, payment of travel-related expenses from AMERSA, the National Safer Supply Community of Practice and the CIHR, and has participated on advisory boards for Health Canada, the Royal Society of Canada, Alberta Health Services and the City of Edmonton.

KDong has received grant funding from the Canadian Research Initiative in Substance Misuse, committee honoraria from the Edmonton Zone Medical Staff Association, payment for conference- and travel-related expenses from the Canadian Association of Emergency Physicians and the Royal College of Physicians and Surgeons of Canada and a medical leadership salary from Alberta Health Services while this work was being conducted.

PML has received grant funding from the CIHR, Alberta Health Services and the Otipemisiwak Mtis Government. He has also received lecture honoraria from the Canadian Association of Emergency Physicians.

These authors declare that they have no other competing interests. All other authors declare that they have no competing interests.

## Authors’ contributions and statement

KAS: Writing—original draft, writing—review and editing.

HB: Data curation, formal analysis, writing—review and editing.

NDG: Writing—original draft, writing—review and editing.

MA: Data curation, formal analysis, writing—review and editing.

KDong: Conceptualization, funding acquisition, writing—reviewing and editing.

PL: Funding acquisition, writing—review and editing.

ST: Funding acquisition, writing—review and editing.

GH: Funding acquisition, writing—review and editing.

PML: Funding acquisition, writing—review and editing.

GS: Conceptualization, funding acquisition, writing—review and editing.

TCW: Funding acquisition, writing—reviewing and editing.

KDm: Funding acquisition, writing—review and editing.

JH: Funding acquisition, data curation, formal analysis, writing—review and editing.

EH: Conceptualization, funding acquisition, supervision, writing—review and editing.

The content and views expressed in this article are those of the authors and do not necessarily reflect those of the Government of Canada.
